# Occurrence of temperature spikes at a wetting front during spontaneous imbibition

**DOI:** 10.1038/s41598-017-07528-7

**Published:** 2017-08-04

**Authors:** Hamed Aslannejad, Alexandros Terzis, S. Majid Hassanizadeh, Bernhard Weigand

**Affiliations:** 1 Department of Earth Sciences, Utrecht University, Utrecht, The Netherlands; 2 Institute of Aerospace Thermodynamics, Stuttgart University, Stuttgart, Germany

## Abstract

It is reported that temperature rises at wetting front during water infiltration into soil. The temperature goes back to the background value after passage of water front. Different explanations have been provided for source of energy causing temperature spike. Some have contributed it to heat of condensation released due to condensation of vapor on “dry” solid surface. Some other stated that the heat of wetting or heat of adsorption is responsible for the temperature rise. In this research, we revisited this issue. First, we provide a comprehensive review about occurrence of temperature spike at a wetting front. Then, we report about experiments we performed on the rise of water in dry paper. Using infrared and optical imaging techniques, we could monitor temperature changes in time and space. For all samples maximum temperature rise occurred at the wetting front. The magnitude of temperature spike depended on paper material, thickness, and liquid composition. It was larger for cellulose-fiber-based paper than for plastic-based paper. For a given paper type, thicker samples showed a larger temperature spike. Adding salt to the water caused reduction of temperature spike. It was concluded that replacement of air-solid interface with water-solid interface releases energy, which causes temperature rise.

## Introduction

Spontaneous imbibition of water is an important process in many natural and industrial porous media. It is observed that water rushes into a relatively dry hydrophilic porous solid despite resistance from viscous drag and even against gravity. Only if the resident phase (e.g. air in the case of unsaturated soil) is put under high pressure, the wetting of the porous medium can be halted. One may pose the questions why spontaneous imbibition occurs and/or which driving force is it that can overcome gravitational and viscous forces. The usual answer is: capillary forces, induced by interfacial tension. In two-phase flow in porous media, there exist indeed three interfaces: two fluid-solid interfaces and one fluid-fluid interface, each with their own interfacial tension.

Commonly a given solid has (a larger) affinity for one of the fluids, called the wetting phase. The other fluid is called the non-wetting phase. The interfacial tension of the non-wetting fluid-solid interface is always larger than that of the wetting fluid-solid interface. Given the fact that the interfacial tension is directly related to the interfacial energy per unit area, we can state that a porous solid filled by the non-wetting phase has a higher energy than when it is filled with the wetting phase, under isothermal conditions. That is why the solid phase would spontaneously imbibe the wetting fluid; the solid-fluids system goes to a lower energy state. The spontaneous imbibition would be stronger, the larger the affinity of the solid phase for a given fluid is. This is clearly reflected in the definition of capillary pressure on both microscale and macroscale. For a capillary tube, the microscale capillary pressure *p* ^*c*^ is given by the Young-Laplace equation:

1 $${p}^{c}=\frac{2}{r}{\gamma }^{wn}\,\cos \,{\theta }$$

where *r* is the tube radius, *θ* is the contact angle, and *γ* ^*wn*^ is the interfacial tension between the wetting and non-wetting fluid phases. This equation can be combined with Young’s equation (equilibrium balance of forces for a contact line) to obtain:

2 $${p}^{c}=\frac{2}{r}({\gamma }^{ns}-{\gamma }^{ws})$$

where *γ* ^*ns*^ and *γ* ^*wn*^ are the solid-nonwetting phase and solid-wetting phase interfacial tensions, respectively. Clearly, the larger the difference between these two interfacial tensions (i.e., the larger the affinity of the solid for the wetting phase), the larger the capillary pressure. A similar link can be established between macroscale capillary pressure and interfacial surface energies. According to Hassanizadeh and Gray^1, 2^, the macroscale capillary pressure, *P* ^*c*^, can be defined as:

3 $${P}^{c}=-\frac{1}{\varepsilon }(\frac{\partial {F}^{wn}}{\partial {S}^{w}}+\frac{\partial {F}^{ns}}{\partial {S}^{w}}+\frac{\partial {F}^{ws}}{\partial {S}^{w}})$$

where *F* ^*αβ*^ is the macroscale specific free energy of *αβ*-interface (i.e., the total free energy of all *αβ*-interfaces within an averaging volume per total area of those interfaces), *ε* is porosity, and *S* ^*w*^ is the saturation of wetting phase. Here, the changes of free energies of bulk phases are neglected. Obviously, for *P* ^*c*^ to be positive, there must be a net decrease of the energy of all interfaces when the saturation of the wetting phase increases. This is the basis of spontaneous imbibition.

This reasoning suggests that during spontaneous imbibition, energy should be released. As we have a closed system, the released energy should result in a (temporary) rise of temperature. This was shown to be indeed the case as early as^3^. Anderson and Linville^3^ observed a temperature spike at a front where a non-wetting phase was displaced by a wetting phase. The rise was of limited duration as the amount of released energy is limited and the fluid arriving behind the front dissipates the generated heat. Early explanation of the observed temperature spike was based on the heat of condensation of vapor on soil grains^3^. It was only later that it was shown that the energy needed for the observed temperature rise is much more than the heat of vapor condensation.

In this paper, first, we provide a detailed review of the literature on the occurrence of a temperature spike at a front where a wetting phase displaces a non-wetting phase. Next, various paper types used in our study and the experimental setup are described. Results are presented and their significance and how they correspond to the results of other authors are discussed.

Although in this work we used water as wetting phase and paper samples as porous medium, however the idea of temperature rise during imbibition of a liquid in a porous material can be used in various areas. The liquid could be in a wide range of solutions, water, blood, urine and the porous material could be paper, soil, a pack of particles. The idea of temperature rise can be used in broad applications; For instance, in case of diagnostic medical kit, a certain temperature rise could be assigned to a concentration or existence of certain component in the liquid.

### Review of experiments on temperature spikes in porous media

**Figure 1:**
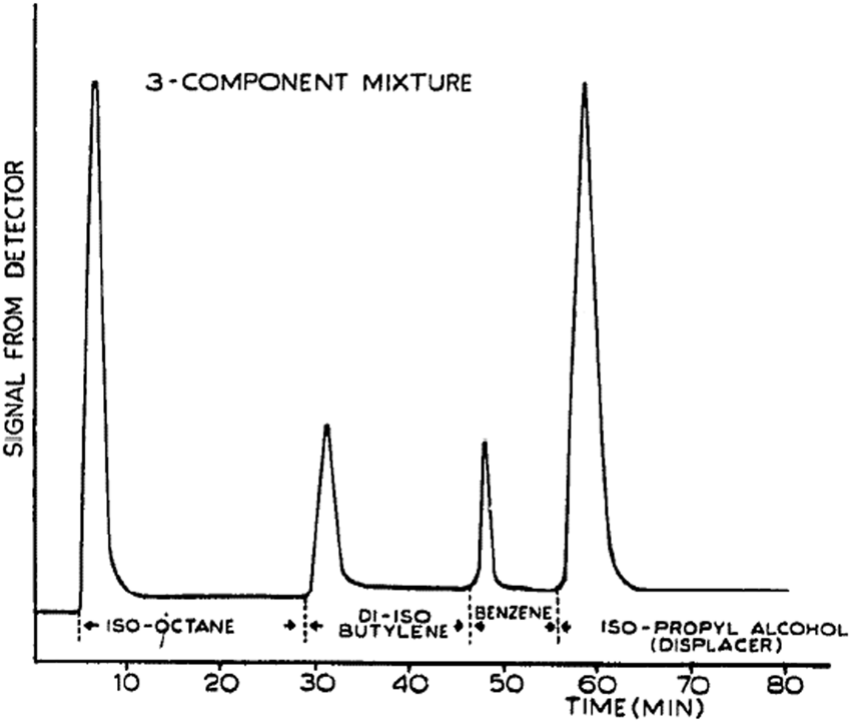
Signal from a thermocouple as a function of time. The temperature spike occurs as the resident liquid is replaced by iso-propyl alcohol^4^.

To the best of our knowledge, Claxton^4^ was the first one to report the increase of temperature of a porous medium upon the arrival of a front. He filled a capillary tube with silica gel saturated with an organic liquid (e.g. iso-octane, butylene and/or benzene) and then the resident liquid was displaced by iso-propyl alcohol. The temperature change was measured by a thermocouple installed near the bottom of the column. The temperature value was not reported as it was a qualitative experiment. But, a clear rise and fall of temperature was shown by the thermocouple (see Fig. 1). Claxton attributed the temperature rise to the preferential adsorption of the displacing liquid on the solid surface. He stated that this “… *is accompanied by the evolution of heat due to a decrease in free energy of the system. This heat is called the heat of adsorption*.”

**Figure 2:**
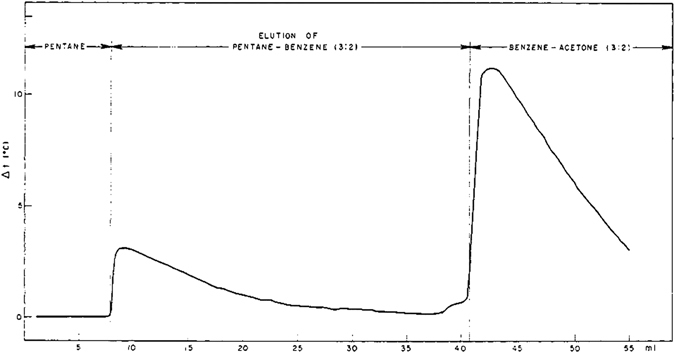
The sharp rise of temperature at the arrival of a liquid front for which the solid phase has a higher affinity, compared to the resident liquid^5^.

Almost simultaneously, a similar concept was developed by Blumer^5^, for use in finding and recording changes in the composition of liquids in liquid-solid chromatographic columns. He measured the change of temperature using a thermistor embedded in a column of silica gel. The column was initially filled by n-heptane, which was then displaced by a mixture of pentane and benzene (3 to 2). After the breakthrough of pentane-benzene mixture, it was replaced by a benzene-acetone mixture. The arrivals of the two fronts were accompanied by temperature rises of about 3 °C and 11 °C, respectively. The temperature returned to the initial value after the front passage (see Fig. 2). Bulmer also attributed such temperature rise to the heat of adsorption.

Anderson and Linville^6^ were the first to perform similar experiments but with water infiltrating into a dry soil sample. They used kaolinite, glass beads, or bentonite as the soil. The porous sample was packed in a vertical plastic cylinder (1.5 cm inside diameter and 6 cm long). At the time of filling of the cylinder, two thermistors were embedded in the sample, one at 5 mm and the other 10 mm from the sample top. Deionized water under a 5-mm falling head was introduced at the top of the sample and allowed to percolate downward. The temperature of the water, sample, and sample holder was brought to 30 °C at the beginning of the experiment in a thermostated chamber which was maintained at constant temperature.

**Figure 3:**
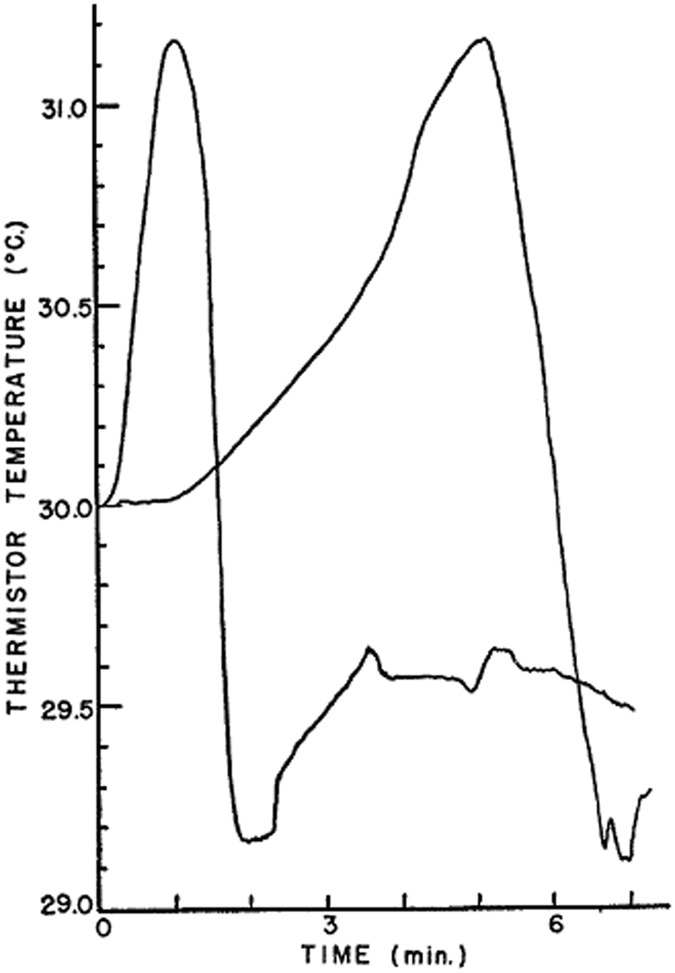
Temperature spikes in dry kaolinite as the water front arrives at two different positions along a soil sample^3^.

They found an increase of temperature at a given location as the water content increased, followed by a temperature decrease (see Fig. 3). They measured a temperature rise of about 0.1 °C for glass beads, 4 °C for kaolinite and about 9 °C for bentonite.

Anderson and Linville^3^ hypothesized that the temperature increase was due to the condensation of vapor on dry solid surface ahead of the front arrival. The released heat was assumed to be the heat of condensation of vapor. The drop in the temperature below its original value was attributed to the cooling of the system due to the evaporation of water at the wetting front.

**Figure 4:**
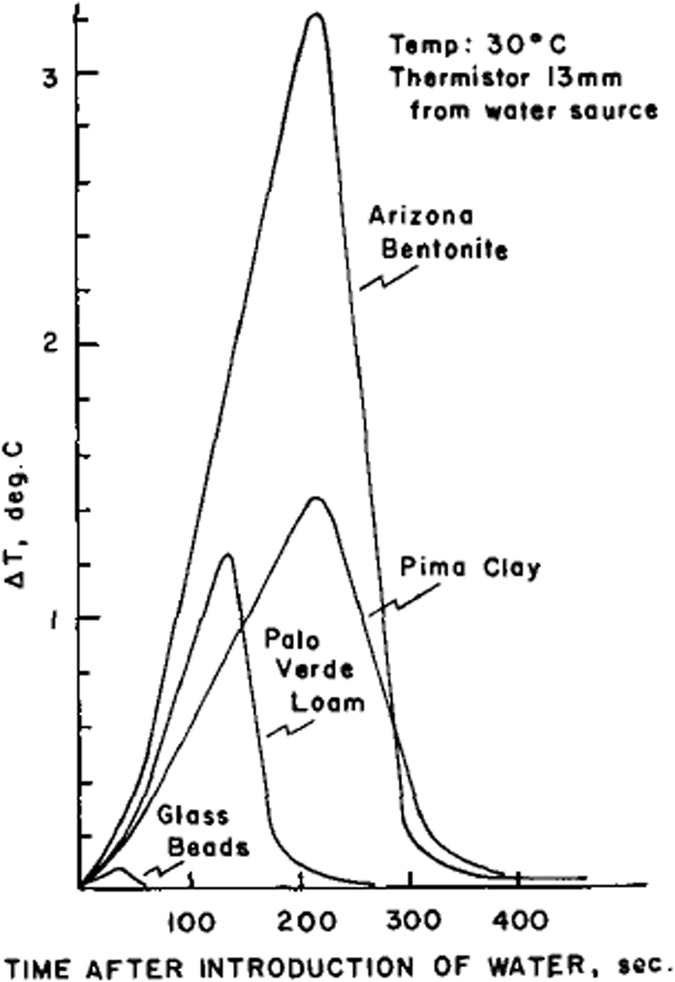
Temperature spikes observed for different types of porous medium (from Anderson and Linville)^6^.

Similar experiments were performed by Anderson and Linville^7^ on more soil types (bentonite, kaolinite, Pima clay, Palo Verde loam, and glass beads) and with different values of initial moisture content. They tried three different types of temperature-sensitive devices: thermistor encased by a tiny glass bead, thermistor positioned in the tip of a hypodermic needle, and thermocouple. They found that the fastest response, and the closest approach to identity with a particle of the various porous media used in their experiments, was obtained with thermistors encased by tiny glass bead. They used glass beads of radius 0.546 mm. This means they were measuring local pore-scale temperature and not the average soil temperature. The ambient temperature of the apparatus was kept at 30 °C by means of a thermostated air bath. For all soil types, Anderson and Linville^6^ found temperature spikes, similar to the results shown in Fig. 4. The curves all have the same characteristics. In all cases, there was a gradual rise in temperature with time elapsed after the water was introduced, followed by a rather abrupt decline in temperature after which the temperature gradually stabilized. However, contrary to the results of Anderson and Linville^3^, this time the temperature did not drop below the original temperature of the medium. In fact, in most cases, it became stable at a level higher than the initial temperature. Anderson and Linville^6^ stated that this “*may be explained by the fact that the wetting process is exothermic and that due to the evolution of the remaining heat of immersion and to thermal conduction, the wetting front need not necessarily be cooler than the initial temperature of both water and medium*”.

**Figure 5:**
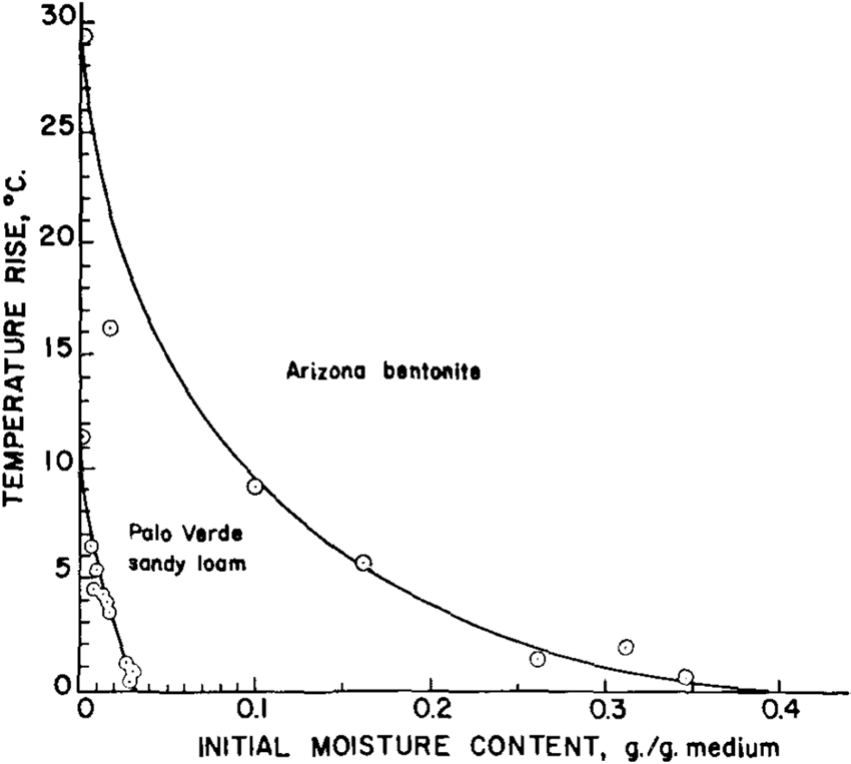
The magnitude of the temperature spike as a function of initial water content for two different soils^7^.

The difference in the magnitude of the temperature spike for different materials was attributed to the difference in their specific surface and affinity for water. It is indeed a fact that bentonite has a much larger specific surface and exchange capacity than glass bead. Consequently, it has a much higher heat of adsorption. The initial water content of the sample was found to affect the magnitude of the temperature spike; it was significantly smaller for higher initial moisture content as seen in Fig. 5 ^8^.

**Figure 6:**
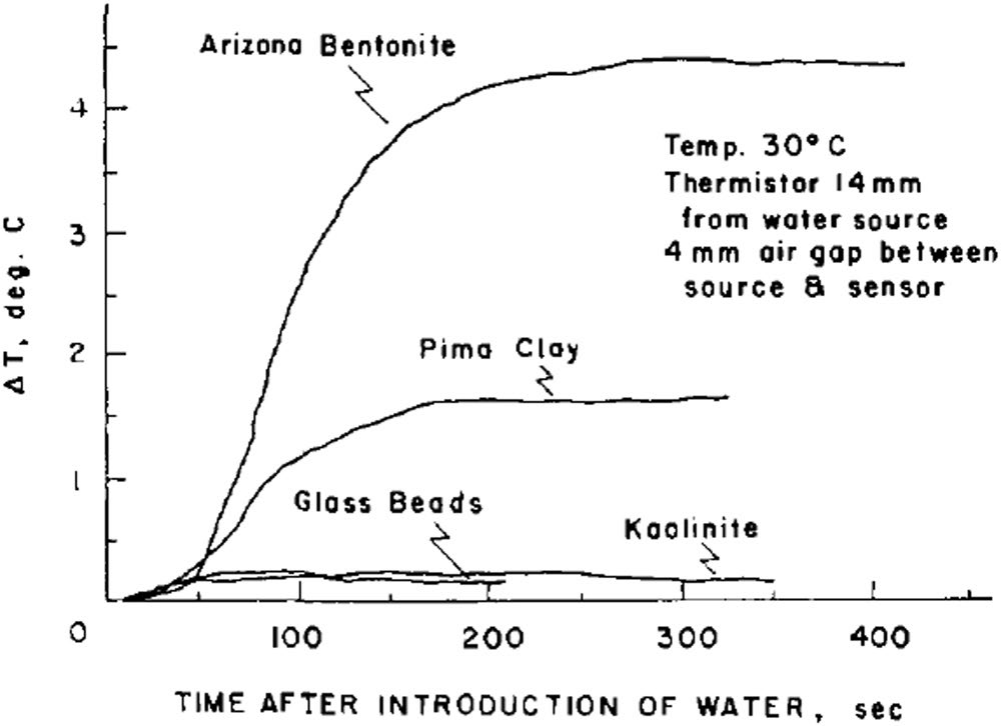
Rise of temperature in a region of soil separated from the water source by an air gap about 4 mm wide. That region was not accessible to the vapor only and not to the liquid water (from Anderson and Linville)^6^.

Anderson and Linville^6^ reiterated the hypothesis that the temperature rise is due to the condensation of vapor moving ahead of the water front. They associated the onset of temperature rise with the arrival of a vapor front at the thermistor site and the moment of sharp temperature drop with the arrival of the liquid front. In order to confirm this hypothesis, they performed two additional experiments. In one experiment, a 0.1N solution of NaCl replaced the distilled water as the infiltrating liquid and compacted kaolinite was used as the porous medium. Their measurements showed that the water content at thermistor site increased during the initial temperature rise, but the chloride ion was not present at the thermistor site. So, this must have been due to condensation of vapor phase. The time that the temperature began to drop sharply coincided with the arrival of the chloride ion, which was in the water phase. In the second set of experiments, an air gap about 4 mm wide was made halfway between the thermistors and the water source by excavating a channel in the porous medium. The plastic specimen holder was coated with silicone grease making it water repellent so that the liquid front would not cross the air gap under the small hydraulic head they employed. The resulting temperature-time curves for Arizona bentonite are shown in Fig. 6. A temperature rises similar to the one shown in Fig. 4 above was found, but there was no drop in the temperature anymore. Anderson and Linville^8^ concluded that the rise of temperature must have been due to the condensation of water vapor that had crossed the gap. Measurement of moisture content of the medium across the gap showed a significant increase. They could confirm that the liquid water did not cross the air gap by visual inspection after the data was recorded.

**Figure 7:**
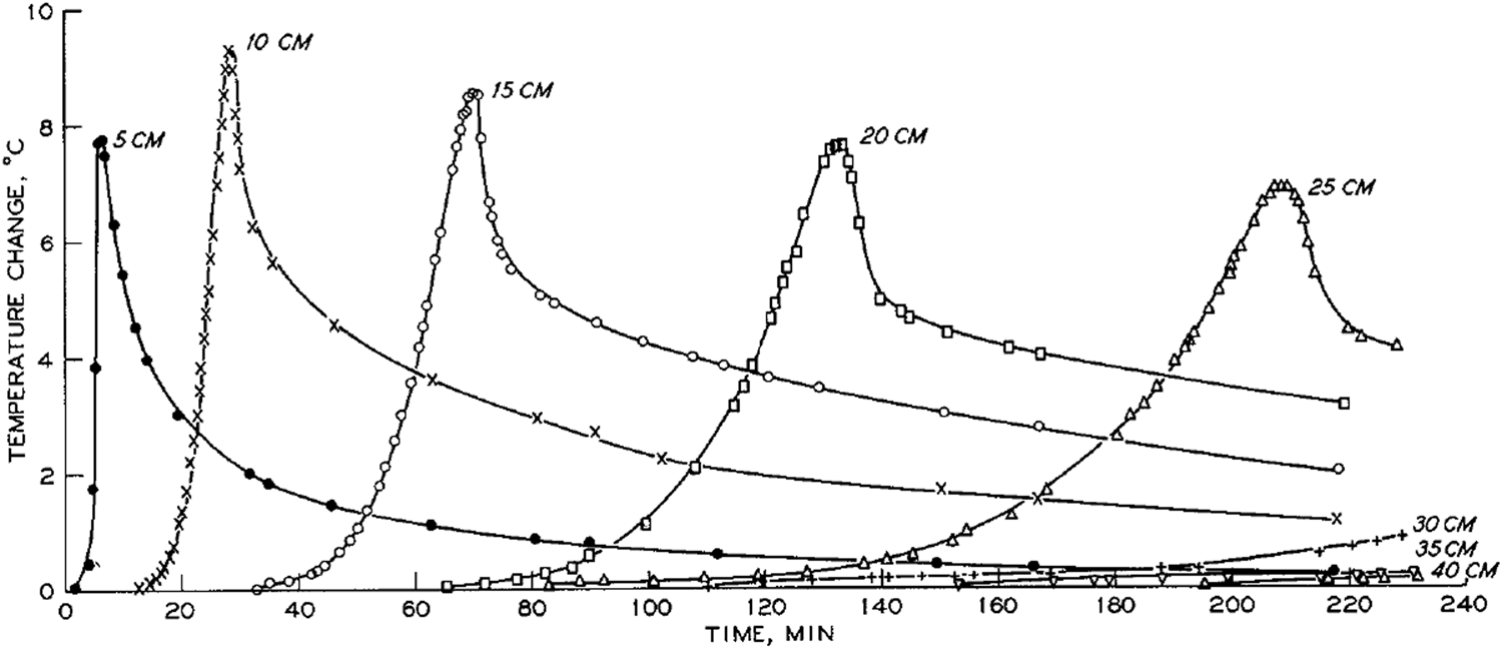
Temperature as a function of time measured at different locations in experiments of Perrier and Prakash^9^.

In experiments by Anderson and coworkers, water flow was downward. So, the flow was not only due to spontaneous imbibition but it was also gravity driven. Horizontal water infiltration experiments, with water flow due to spontaneous imbibition only, were performed by Perrier and Prakash^9^. Also, they measured not only temperature but also air humidity in the soil. They prepared two columns, both filled with Flanagan silt loam; air dried for one column and oven dried for the other column. Typical results are shown in Fig. 7. As with earlier experiments, the temperature rose as the water approached a given position, and decreased after the passage of the front. But, contrary to experiments of Anderson and coworkers, it stayed above the original temperature of the column. Also, the magnitude of the spike became smaller with increasing distance along the column.

**Figure 8:**
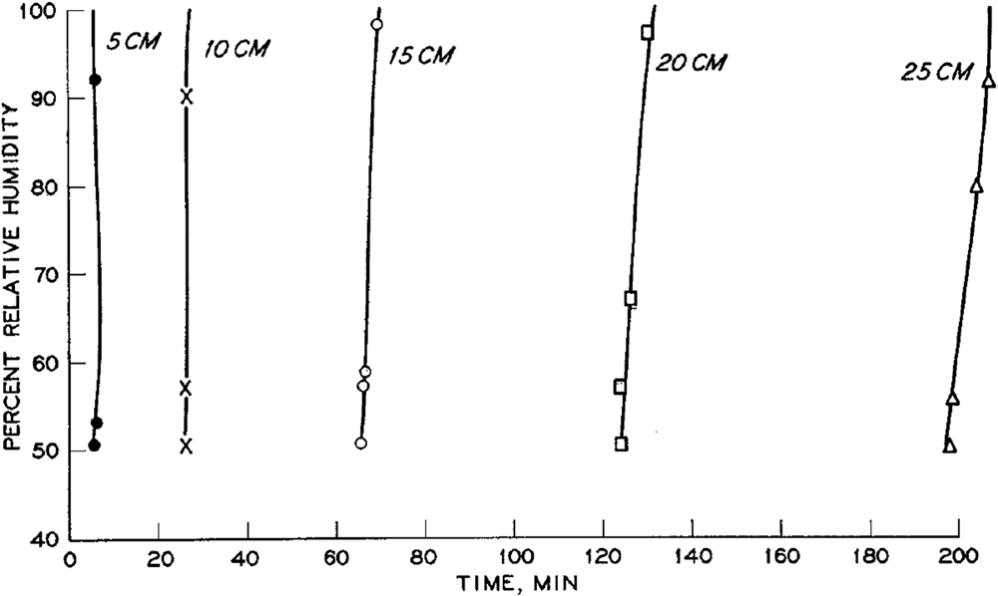
Instantaneous increase of relative humidity at various positions along the column (from Perrier and Prakash)^9^.

**Figure 9:**
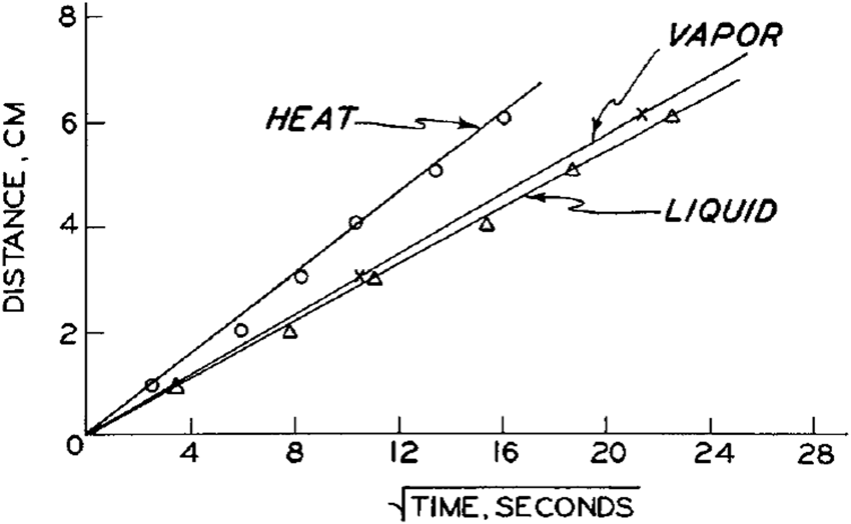
Position of temperature, humidity and water content fronts (from Perrier and Prakash)^9^.

Measurements of relative humidity transducers showed that the air humidity increased almost instantaneously from background value to 100% relative humidity (see Fig. 8). It is clear that the temperature starts to rise much earlier than the rise of the air humidity. Perrier and Prakash^9^ considered the first inflection point of the temperature profile as the position of temperature front. Because the horizontal column was made of Lucite, they could also follow the macroscopic liquid water front. They observed that the thermal peak (site of greatest temperature gradient) coincided exactly with the position of the liquid front. “*Consequently*,” they wrote, “*the thermal peak was taken to be the location of the liquid front for the data presented*.” A plot of the locations of three fronts as a function of time is shown in Fig. 9. It is evident that the vapor front arrived only slightly ahead of the water front. But, the temperature rise occurred much earlier than the change in humidity and moisture content. Perrier and Prakash^9^ did not provide any explanation for this phenomenon.

Perrier and Prakash^9^ concluded the following from their experiments: “*These data show that the advance of a wetting front involves several processes: as dry soil is wetted, a large amount of heat is evolved; (b) evaporation during the wetting process supplies a vapor phase; (c) as liquid moves into dry soil, the vapor phase moves as a front immediately ahead of the wetting front; (d) a large portion of the heat evolved moves as a front well in advance of both the vapor and liquid fronts; and (e) the heat evolved subsequently heats the liquid front; however, due to evaporation and the thermal conductivity of water, the wetted soil behind the liquid front is cooled*.” So, they suggested that the heat is released due to the wetting of soil and evaporation occurs afterwards as a result of this heat generation. This is opposite to the assertions of Anderson and Linville^6^, who stated that the evaporation occurs spontaneously first and the heat is generated due to the vapor condensation on soil grain surfaces.

**Figure 10:**
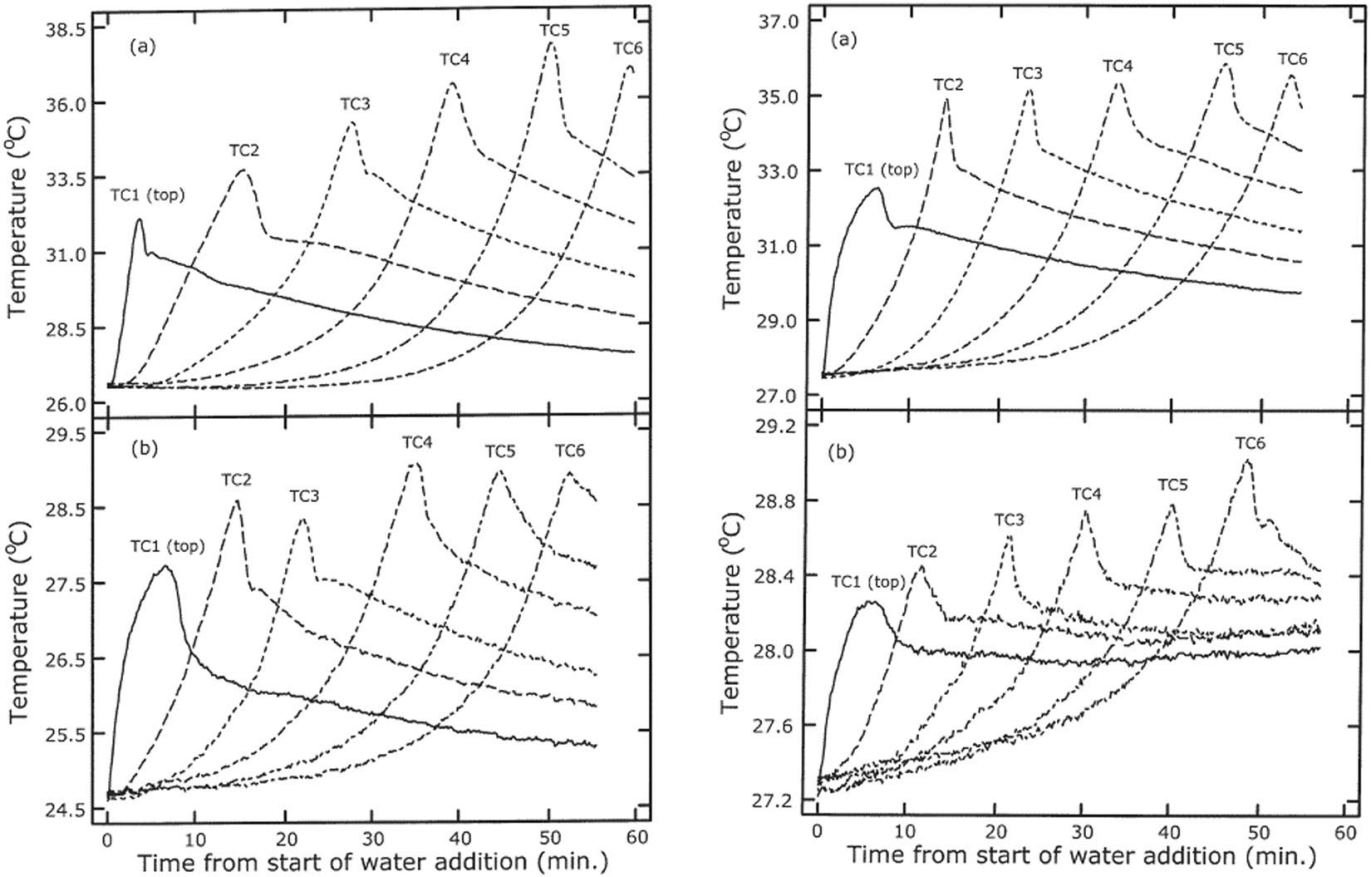
Temperature as a function of time measured at different locations in experiments of Prunty and Bell^10^. The two plots on the left are from columns containing silty clay soil (**a**) dry and (**b**) with 0.04 g/g. Plots on the right are for loamy soil.

The hypothesis of Anderson and Linville^6^ was also challenged by Prunty and Bell^10^, who performed experiments similar to theirs with water flowing downward into (partially) dry soil (see also Prunty^11^). They used either Fargo silty clay or Glyndon loam, at different initial moisture values (0.00, 0.02, 0.04, and 0.06 g/g). They measured the temperature using thermocouples at six different locations along the column. For dry soil, they found temperature spikes of 5 °C to 11 °C, depending on soil type, initial moisture content, and the position along the column. Typical results are shown in Fig. 10. The magnitude of temperature spikes became larger along the flow direction; this was contrary to findings of Anderson and Linville^3^, who didn’t find a change and Perrier and Prakash^9^ who found a decrease along the flow direction. However, similar to Anderson and Linville^8^, they found that the temperature rise was significantly smaller for partially wet sand. They did not observe any cooling of the medium; in any of the cases. In fact, the temperature dropped back but remained above the initial temperature. Using physical properties of the soils used in their experiments, Prunty and Bell^10^ estimated the expected temperature rise when the soils would get saturated from the oven-dry condition. They considered two different thermodynamic state changes: one when water vapor condenses on the dry surface of soil grains (with released energy being called heat of adsorption) and the other when liquid water completely covers the dry grain surfaces. They referred to the latter as the heat of wetting. They clearly showed that while the heat of wetting alone is sufficient to cause temperature rises measured in their experiments, heat of adsorption can cause only small temperature rises. They concluded that heat generated by the wetting of soil grains by liquid water is the main source of a temperature peak at the wetting front. This was in line with the conclusion of Perrier and Prakash^9^.

**Figure 11:**
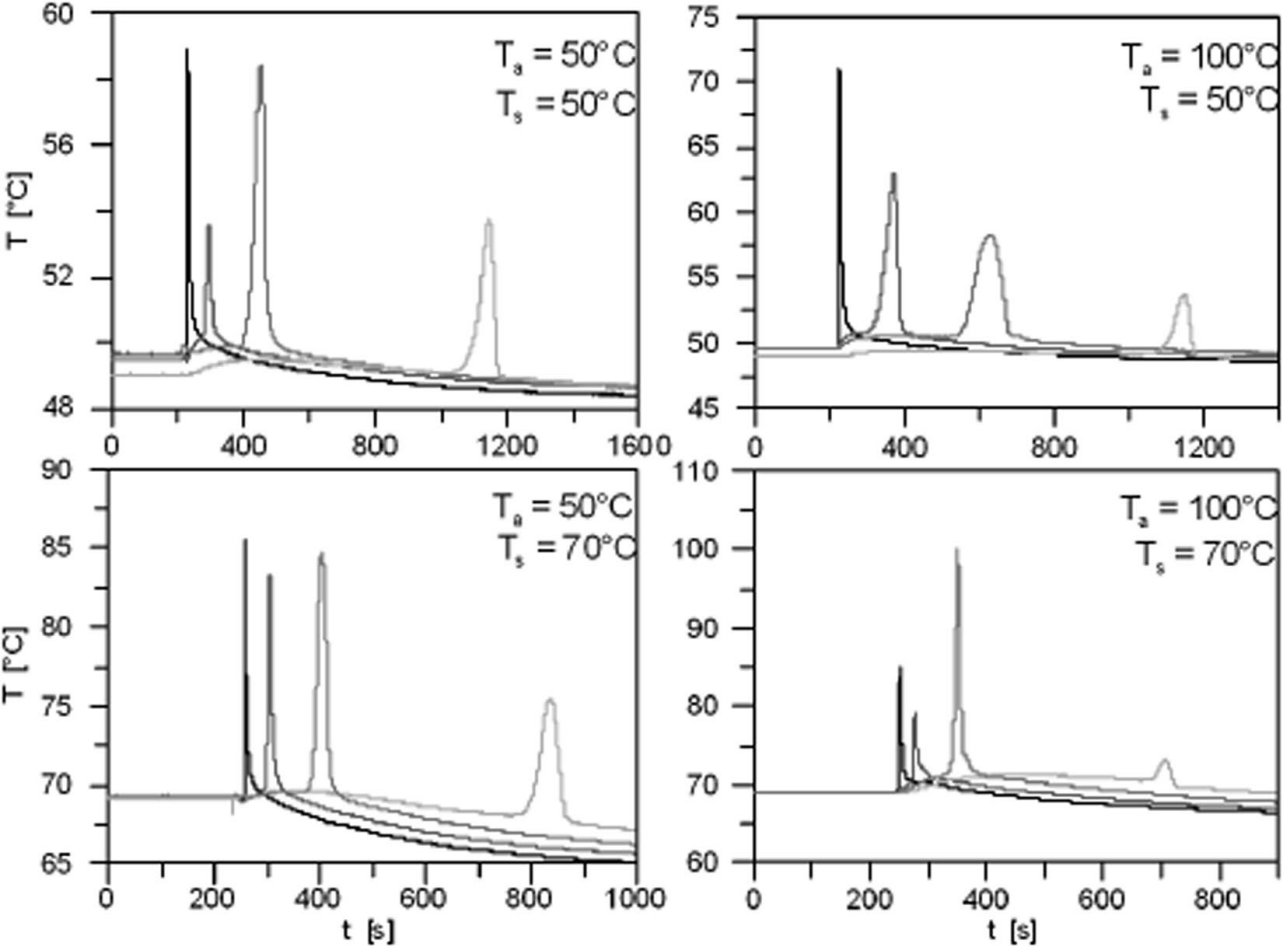
Temperature as a function of time in experiments of zoldak- Nowak *et al*.^12^. Two diagrams in the right were done on Zeolite pickings treated in 100 °C and ambient temperature 50 and 70 °C. Diagrams in left, Zeolite treatment in 50 °C and ambient temperature 50 and 70 °C.

Recently, spontaneous infiltration experiments were performed by Zoladek-Nowak *et al*.^12^, with water rising into a column by capillarity. They introduced water into the bottom reservoir of a column of dry natural zeolite grains. Water rose spontaneously in the column. The water content change in the column was monitored using neutron radiography and the temperature was recorded by thermocouples placed at four different elevations in the column. They performed many experiments at various ambient temperature and different treatments of zeolite grains. Experiments were carried out at five different constant ambient temperatures, ranging from 30 °C to 70 °C. Zeolite grains were treated at three different drying temperatures (50 °C, 100 °C, and 150 °C) for two to five weeks before packing them into the column. Typical results are shown in Fig. 11. They found temperature spikes of 3 °C to 40 °C in various experiments, as the wetting front passed positions along the column. The largest amplitudes were for cases of higher ambient temperature and/or when zeolite grains had been treated at higher temperature. They explained that zeolite becomes more hydrophilic under those conditions. As a result, there should be more energy released, leading to a larger temperature spike.

This review shows that some of the early theories about the source of heat for temperature rise at a wetting front are questionable. In particular, the idea that the temperature rise is due to the heat of condensation of water vapor ahead of the wetting front does not hold. In fact, if temperature rise were due to the water vapor condensation on solid surface of the porous medium, it should have been observed not only at the water front but also everywhere else in the porous medium; the vapor can diffuse relatively fast in the pore space and should therefore condensate everywhere. That has not been observed. Moreover, if water vapor would wet the solid surface and reduce its surface energy, then there would remain no driving force for spontaneous imbibition to occur. That is, once the vapor diffuses into a porous medium and wets the pore surfaces, there is no thermodynamic reason for water to enter the medium, as vapor competes with its own liquid in wetting the solid surfaces. Evidence for this statement can be found in a neat experiment reported by Bangham and Razouk^13^. They found that a drop of methyl alcohol spread rapidly when it was placed on freshly-split mica, when the experiment was done in air. But, when air was replaced with the saturated vapor of methyl alcohol, it did not do so; the spread film of methyl alcohol formed in air immediately gathered back into lenses when it was brought under a jet of the supersaturated vapor of the alcohol. This means that vapor condensation on surfaces of pores should actually hamper spontaneous imbibition, which we know it does not occur. So, the only plausible explanation for the source of energy that causes an increase in temperature of the porous medium and the rise of water in the medium, against gravity and viscous forces, seems to be the heat of wetting; i.e., the energy released due to the spontaneous replacement of air-solid interface by the water-solid interface.

## Methods

### Paper samples

**Figure 12:**
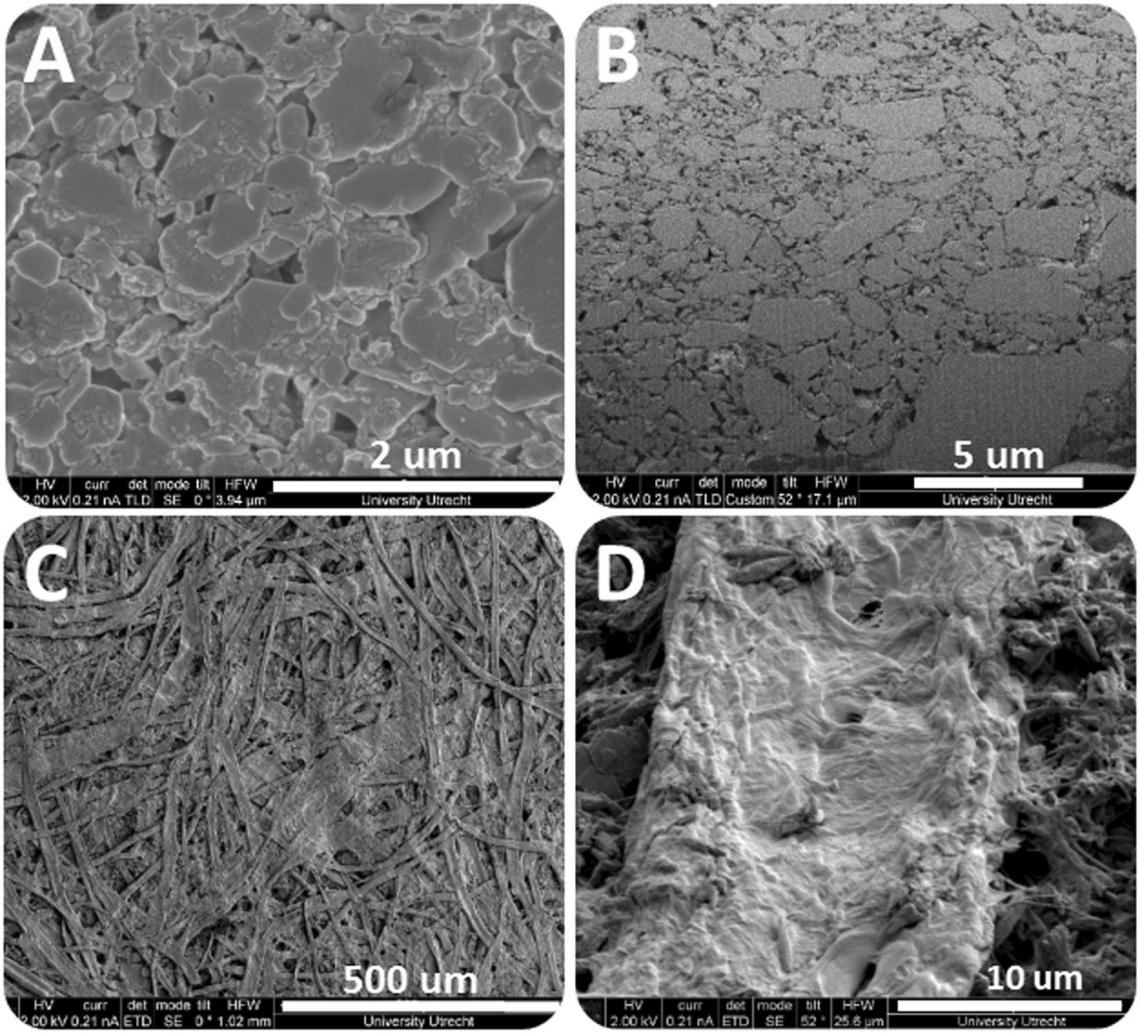
SEM images of surface and cross section of a coating layer (**A,B**, respectively), fibrous layer surface (**C**) and surface of a single fiber (**D**).

Six different paper types were used in this work including coated and uncoated papers. Five papers were made of natural cellulose fibers and one from plastic fibers. The fiber material affects the penetration of liquids into the paper significantly, even when overall porosities and mean fiber diameters are almost the same. For example, cellulose fibers consist of a bundle of fibrils^14^, which are themselves porous. Also, their surface is rough and contains Nano-channels (see Fig. 12D). But, plastic-based fibers are usually not porous and have smooth surfaces. So, water can enter plastic-based papers only via pores formed between the fibers. While in cellulose paper, water moves not only in large pores formed between the fibers, but it also gets imbibed into individual fibers and seep into nano-channels on the surface of fibers. Another important difference between natural and plastic fibers is in the wettability of their surfaces. Cellulose fibers have hydrogen bonds in their structure, so they are hydrophilic while plastic fibers are usually hydrophobic^15^. That will have a major effect on capillary suction and the amount of released heat. Figure 12 shows micro structures of samples of paper coating (A. surface and B. cross section) and fibrous layer (C), and the roughness on the surface of a cellulose fiber. These images were obtained by Scanning Electron Microscopy (SEM).

**Figure 13:**
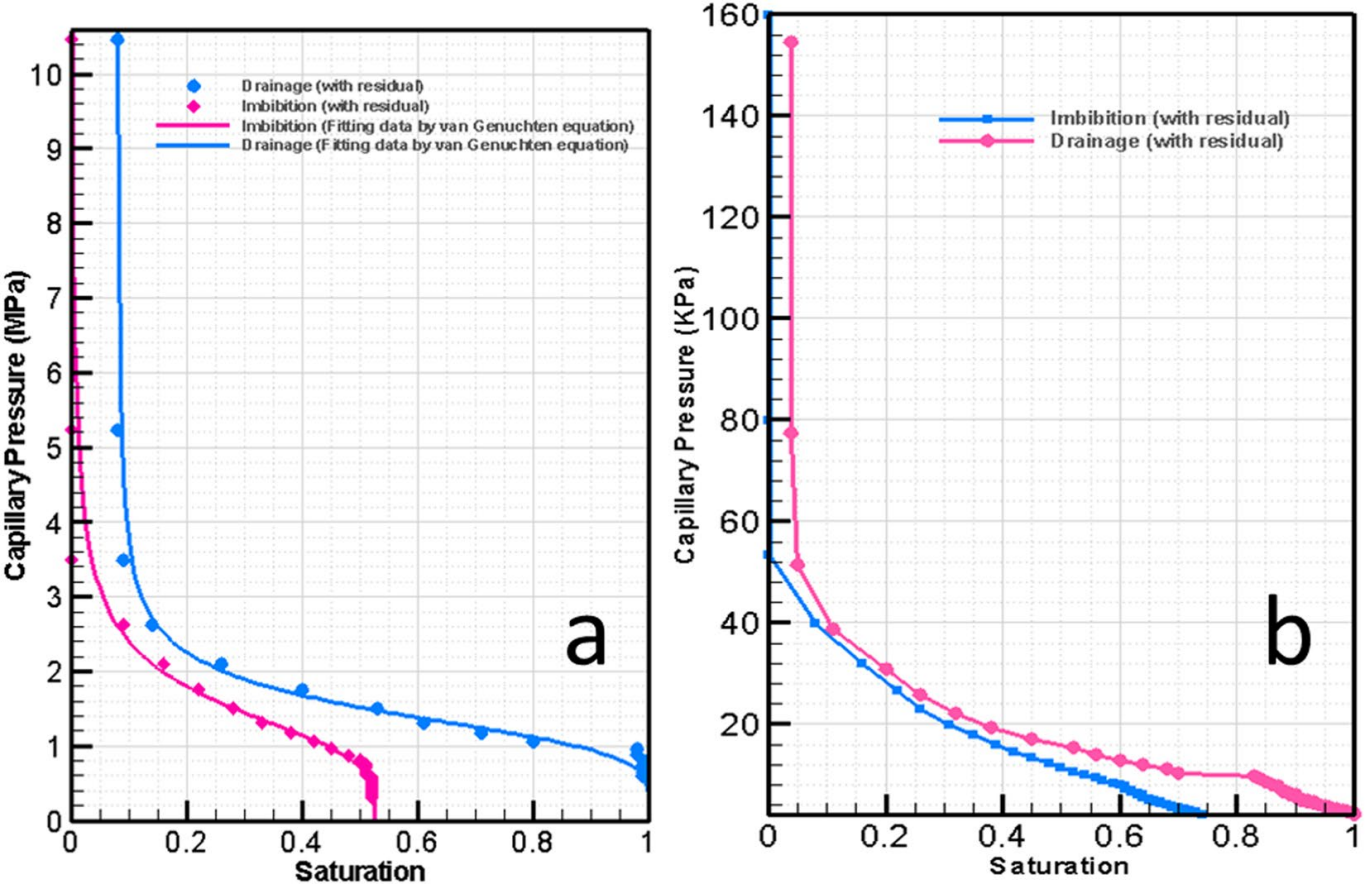
Capillary pressure curves for; (**a**) coating layer and (**b**) fibrous layer^16^.

Specifications of the six paper types used in this study are given in Table 1. The Ziegler paper is like the regular papers which are used in office printers. It is an uncoated cellulose fibrous paper with a thickness of 120 µm, mean pore size of 10 µm, and a porosity of 52%. The coated paper, Magno gloss, is made of a base fibrous layer, 65 µm think, with coating layers on both sides. Each coating layer is 10 µm thick; so, the total thickness of the paper is 85 µm. The coating layer is made of CaCO_3_ powder, with mean pore size of 180 nm^16^. The base layer is made of cellulose fibers with mean fiber diameter of 20 µm. Figure 13 shows capillary pressure-saturation curves for the fibrous and coating layers; obtained by simulation (ref. under review, will be added later).

**Table 1:** Paper samples characteristics.

Paper type	porosity	Mean pre size	Layer(s)’s material	Thickness
Ziegler	52%	10 µm	Cellulose	120 µm
Coated paper (Magno glass)				
Coating layer	34%	180 nm	CaCO_3_	10 um
Fibrous layer	50%	(2×)10 um	Cellulose	65 um
RL 200	50%	12 µm	Cellulose	260 µm
RL professional	48%	12 µm	Cellulose	340 µm
Plastic based	65%	16 µm	Plastic	220 µm
Prematex	60%	20 µm	Cellulose	300 µm

In addition to the Ziegler paper, we used two similar but thicker papers; RL 200 and RL professional. The material type is the same for these two papers, but the thicknesses are 260 µm and 340 µm, respectively, for RL 200 and RL professional.

Some fibrous base layers contain a filler material, made of very fine powder (average grain size of about 1 µm). The most common paper fillers are ground calcium carbonate, kaolin, precipitated calcium carbonate, talc, and titanium dioxide. The amount of fillers varies from none to at least 30% of the whole furnish. The main reasons to use them are their low cost, compared to fiber, and improvement of optical properties of the print (Brightness, Whiteness and Color) of the final product. Fillers can also improve surface properties of paper and thus have a positive effect on the printability of the final product^17^. Among paper samples, Plain paper, RL 200 and RL professional, have filler materials inside their fibrous layer.

The fibrous layers of Ziegler, RL 200 and RL professional papers are impregnated with mineral particles (added as fillers). We also used a sample of Prematex paper, which consists of only fibers with no mineral particles added. Its porosity is higher (60%) relative to Ziegler paper (52%) and it has a much larger thickness.

### Experimental setup and procedure

**Figure 14:**
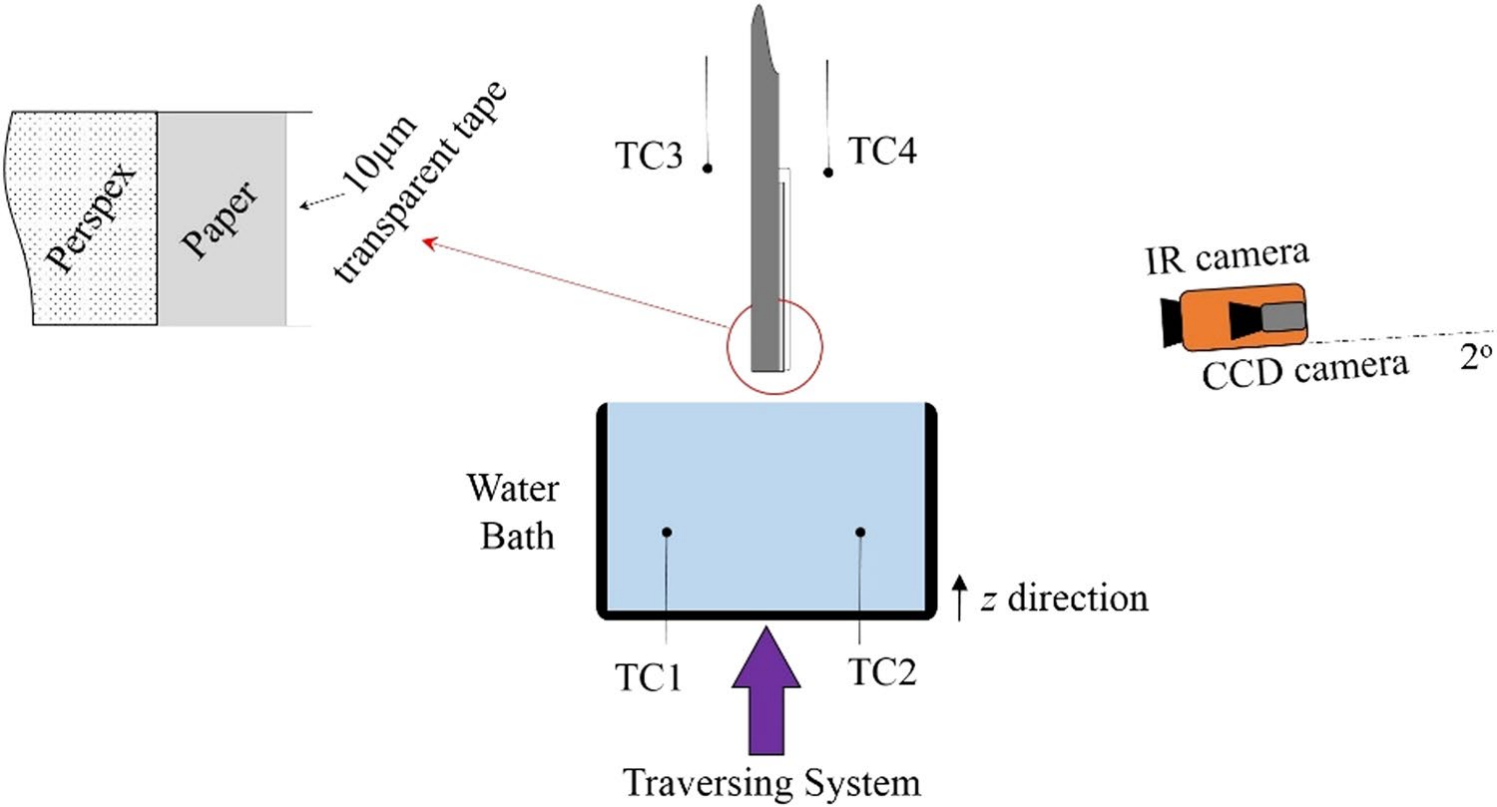
Schematic of the experimental setup.

Four strips were cut from each paper type in order to quadruplicate the data in a single experiment. Each strip was 45 mm in length and 10 mm in width. As the fiber orientation influences the layer structure and consequently water movement, all samples were cut in the same direction (along length). In order to prevent the evaporation of water from the wet paper surface, the samples were completely covered and pressed onto a Plexiglas plate by a 62-µm-thick clear tape, which was also transparent to infrared radiation (see inset in Fig. 14). The lower edge of the tape and the paper sample were carefully trimmed with a surgery blade to be exactly aligned with the lower edge of the Plexiglas material.

The Plexiglas plate was kept in room temperature overnight. It was fixed in the vertical position to an experimental rig. In order to increase the quality of thermal images, the Plexiglas surface was covered with a matt black adhesive ensuring a high emissivity value of 0.98. The emissivity of dry and wet paper areas was determined based on the ambient and liquid bath temperatures. The resulted emissivity for both areas was very similar, and hence, a single value of 0.9 was considered for the whole imbibition process.

A schematic representation of the setup is shown in Fig. 14. A constant-temperature water bath was placed on a traversing stage under the Plexiglas plate. The bath was filled with distilled water. The imbibition process could be started by raising the water bath slowly until the water surface would touch the lower edge of Plexiglas and the paper strip. The temperature of the bath was set at 0.2 degrees lower than ambient temperature in order to ensure that no heat was transferred from the water to the paper samples. Hence, any temperature variation of the paper could be attributed to the imbibition process only. The temperature of the distilled water was measured with two K-type thermocouples (TC1 and TC2 in Fig. 14). The ambient temperature was monitored by two thermocouples positioned on the two sides of the experimental rig (TC3 and TC4) as shown in Fig. 14.

Optical and thermal cameras were installed in front of the Plexiglas plate. The spatial distribution of temperature was recorded continuously by a FLIR-SC7000C IR camera with high sensitivity, and noise levels as low as 20 mK. The IR camera had an optical lens of 25 mm with a maximum resolution of 640 × 512 pixels, which resulted in approximately 45 pixels/(mm)^2^. In order to increase the quality of the infrared measurements, the backside of the experimental test rig was covered with a black curtain in order to prevent any reflection and radiation influence of the surroundings on the IR camera display. However, the top of the test rig was open and normal room illumination was provided. In addition, the CCD videos were taken with a relatively large exposure time in order to increase the contrast of the images. As the tape was transparent to infrared radiation, the IR camera actually measured the temperature of the paper surface just below the tape.

The imbibition of the water into the paper was recorded with a high definition RGB camera, model UI-3360CP, connected with a frame grabber to a 16GB-RAM computer. These hardware capabilities allowed real time image saving. Thus, images with a high spatial resolution of approximately 55 pixels/(mm)^2^ could be acquired while a KOWA LM6JC lens of 16 mm eliminated image distortion. Note also that the CCD camera was used in the monochrome mode in order to increase the contrast and better distinguish between wet and dry paper surfaces.

**Figure 15:**
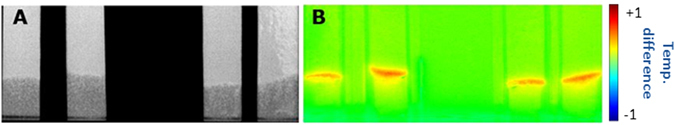
Examples of acquired images: (**A**) optical and (**B**) thermal.

Both cameras were set to a constant frame rate typically varied between 5Hz and 15Hz depending on the paper sample and the associated imbibition speed. The imbibition processes typically lasted from about 2 to 12 minutes. The first frame of CCD camera was determined by small reflections of the paper on the water surface observed in the optical video, which was also used as a reference to define the beginning of the thermal recording. Hence, the start of the thermal and optical videos was synchronized with an accuracy of one frame. An example of optical and thermal images at a given instant is shown in Fig. 15.

To start the imbibition experiment, the water bath was raised using a precise traversing system. As soon as the water covered a few mm of the Plexiglas plate holder and the paper samples, and the imbibition process started, we fixed the position of the water bath.

The experimental set up presented above has been recently employed by Terzis *et al*.^18^ in order to investigate the imbibition of a number of other liquids into paper.

## Results

### Positions of temperature and wetting fronts

**Figure 16:**
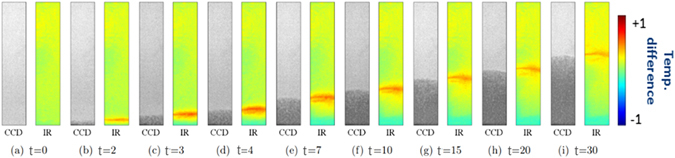
Optical and thermal images of RL 200 paper during spontaneous imbibition at various times (sec).

**Figure 17:**
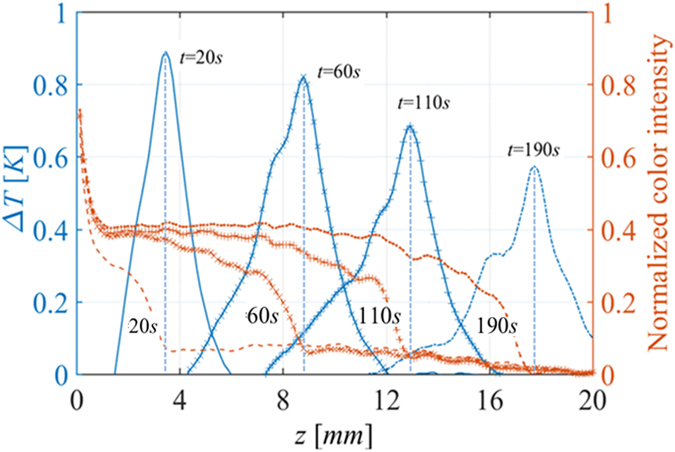
Profiles of saturation and temperature along a sample of RL 200 paper at different times (The measured positions were in respect to the lower edge of Plexiglas plate). Vertical dashed lines indicate the positions of temperature and wetting fronts.

Figure 16 shows optical and thermal images at various times during the spontaneous imbibition process, for RL 200 paper. Similar images were obtained for all other paper types. The dark-colored region in black-and-white images from CCD cameras shows the wetted part of the paper. Based on these images, profiles of gray color intensity (indicative of water distribution) and temperature at different times were obtained and are plotted in Fig. 17. It is clear that the maximum temperature at any given point was reached when the color intensity visibly started to increase there; we refer to this point as the water front (shown by a vertical dashed line in Fig. 17). The increase of temperature starts even earlier because it is known that precursor water films move ahead of a water front; thus, water film could cover solid surfaces and cause the release of energy and increase of temperature before any sign of moisture increase visible to the CCD camera. The temperature started to decrease and return to the ambient value as more water, which was slightly below the ambient temperature, arrived and almost fully wetted the paper.

**Figure 18:**
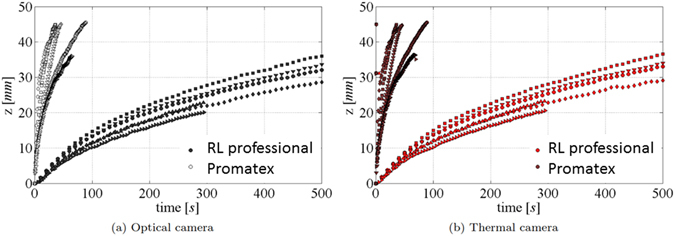
Imbibition height as a function of time for the optical and thermal image.

A plot of the positions of wetting front and temperature front (where the maximum temperature was reached) as a function of time is shown in Fig. 18a,b, respectively, for RL professional and Prematex papers. In each case, there are four curves corresponding to the four samples of the same paper. We did not plot wetting and temperature front positions in a single graph because they would coincide and it would be hard to distinguish them. The wetting front speed was higher in the case of Prematex paper than for RL professional paper because of its significantly higher permeability.

**Figure 19:**
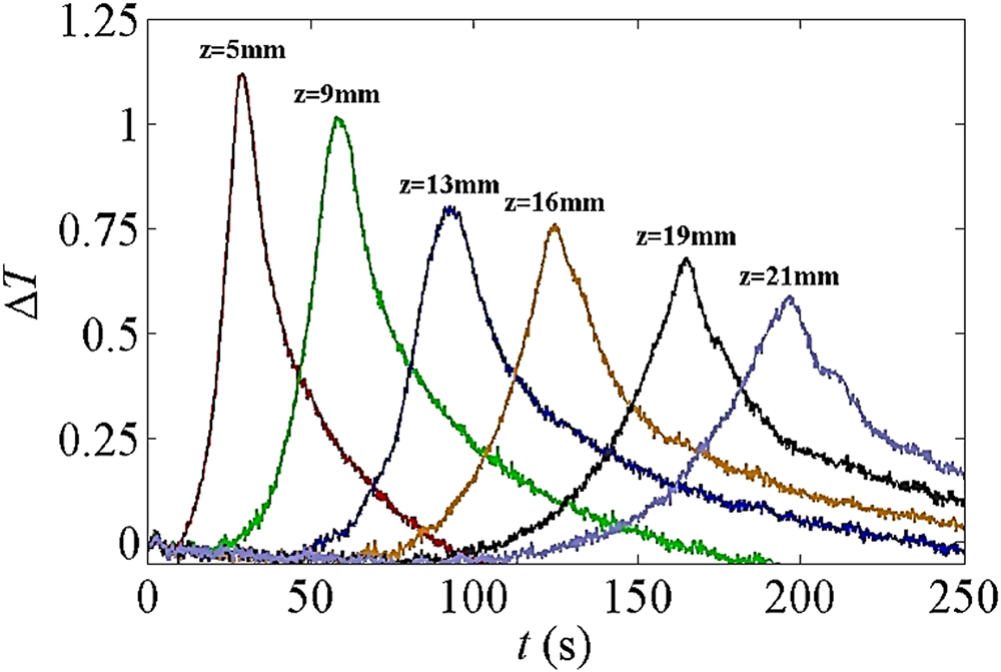
Temperature rise at various imbibition heights over time.

We know that as water rises up in a porous medium spontaneously, the wetting front will become more diffuse and the corresponding maximum saturation will be lower. This means that the amount of wetted air-solid surface per unit volume becomes less and less as the wetting front rises. This is reflected in the change of temperature with time at various positions along the paper, shown in Fig. 19. The temperature spike at higher positions is wider (because the water front is more diffuse) and its magnitude is lower (as relatively less energy is released).

### Effect of paper thickness and paper type

**Figure 20:**
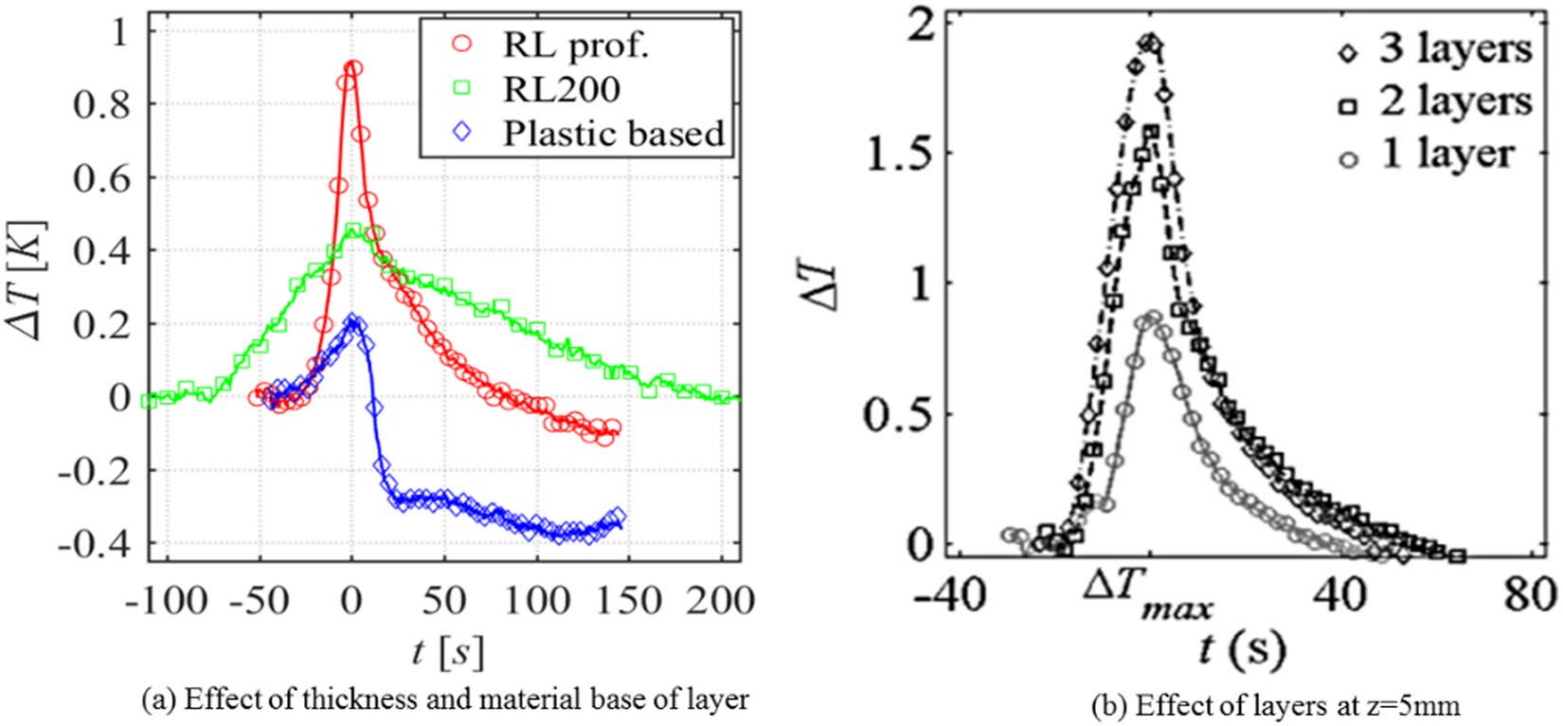
Effect of thickness and paper type on temperature rise.

In our setup, the released energy goes into warming up not only the paper sample but also the tape covering the paper and the Plexiglas plate behind the paper samples. So, there was significant loss of heat to the tape (and thus the air) and the Plexiglas plate. This heat loss is more or less the same for a thin paper or a thick paper. Now, for a thicker paper (or more layers of paper), there is more energy released. However, there is also a proportionally larger mass to be heated up. Nevertheless, as the heat loss remains the same, the ratio of heat loss to the released energy will decrease for thicker paper samples. So, relatively speaking, there is more energy available to thicker papers or for more layers of the same papers and this should cause a bigger rise of the observed temperature. This could be confirmed by comparing results of temperature change for RL 200 and RL professional papers. They are the same paper type but with different thicknesses, namely 260 µm and 340 µm, respectively (see Table 1). We also performed experiments with one, two, and three layers of the RL professional paper. Thus, the total thickness of the sample in those experiments was 340 µm, 680 µm, and 1020 µm, respectively. The plots of temperature change with time for RL 200 and RL professional papers at the position z = 5 mm are shown in Fig. 20a and the same for 1, 2, and 3 layers are shown Fig. 20b. It is clear that the magnitude of temperature spike is significantly larger for more layers of the same paper or for a thicker paper.

We also investigated the influence of the paper type by performing experiments on a sample of plastic-based paper. The sample was 220 microns thick and was made of a hydrophobic non-cellulose-based material. As the solid phase is hydrophobic, it has a large solid-water interfacial tension. That means that we should expect significantly less energy released, and thus a lower temperature spike, at a wetting front, compared to a cellulose-based paper. The plots of temperature change with time for RL professional, RL 200, and plastic-based papers at the position z = 5 mm are shown in Fig. 20a. It is evident that the magnitude of temperature spike for the plastic-based paper was significantly lower than for the cellulose-based papers.

### Effect of water properties

**Figure 21:**
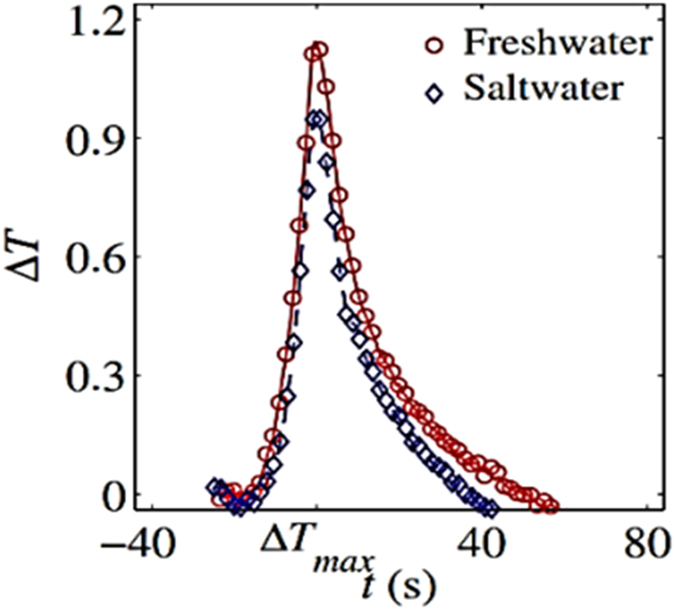
Temperature rise during water spontaneous imbibition and effect of surface tension at z = 5 mm.

As the temperature spike is completely controlled by interfacial energies, we investigated the effect of changing water properties in order to reduce its affinity to the solid phase. We did this by dissolving salt in water creating a salty solution with the concentration of 120 gr/l. The solid is known to be less wetting to saltwater compared to fresh water. In other words, the interfacial energy of salt water-solid surface is larger than that of fresh water-solid interface. Thus, one would expect less heat to be released when air-solid interface is wetted by salt water. This was clearly confirmed by the plot of temperature change with time shown in Fig. 21; the magnitude of temperature spike for the salt water was significantly lower than for fresh water.

### Evaporation and condensation of water

**Figure 22:**
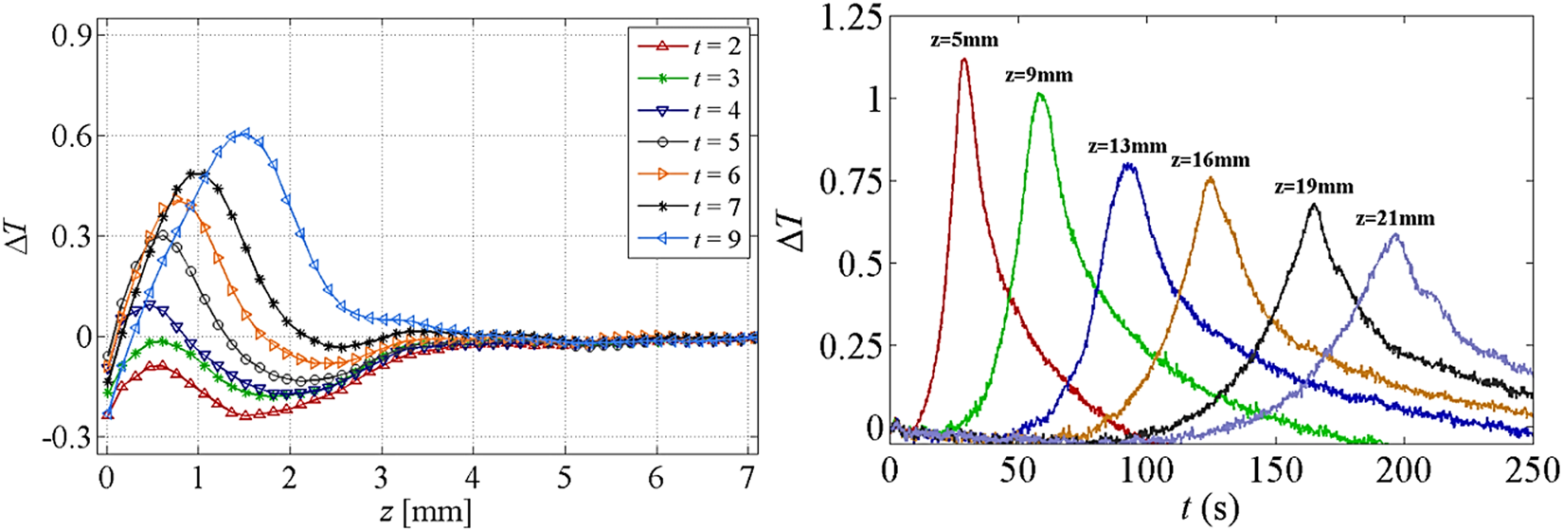
Averaged temperature distribution at various time frames at very initial imbibition stages (left).

As mentioned before, one of the early theories proposed for explaining the temperature spikes was based on the condensation of water vapor ahead of the wetting front (*cf*. Anderson and Linville^3, 6^). But, of course, water has to be evaporated first, and water evaporation is expected to be accompanied with cooling of the system. We looked for such a cooling effect in our observation. Indeed, at very early imbibition stages, *i.e*., very low imbibition heights (3–4 mm above the water source), the IR images showed a small temperature drop, which was still resolvable with IR camera. Figure 16 shows IR images for various time frames. Also, in Fig. 22, we show the temperature profile with distance at very early times. Both images show that local cooling due to evaporation occurred at the very early imbibition stages. However, no cooling was observed as the imbibition process propagated.

This can be attributed to two reasons:

- The temperature rise caused by wetting of solid surface overcomes the evaporation cooling.
- The air ahead of the liquid front becomes ‘saturated’, and hence, no more evaporation occurs.

As it can be seen in the videos of our experiments (see related file in Supplementary Material section), the rise of temperature stopped after the water front stopped. But, one would expect the evaporation of water and its condensation to occur far above the wetting front. So, the temperature rise should have been observed in the upper part of the sample even after the wetting front stopped. This was not the case.

## Conclusion

There exist three interfaces in a partially-saturated porous material: air-solid, solid-liquid, and liquid-air. The surface energy of solid-air interfaces is known to be much larger than that of the water-solid interface. The spontaneous imbibition of water into a medium, such as dry paper, occurs due to the net decrease in interfacial energies of those three interfaces. The released energy is turned into heat which causes a temperature spike of the porous medium (water plus paper fibers). The magnitude of the spike depends on material, thickness, and liquid composition. Our results show that the maximum temperature at any given location occurs exactly at the time that water front arrives. Effects of various factors such as paper type, its thickness, number of layers, and liquid composition were investigated. A more extensive study of effects of different liquid on temperature rise was done recently by Terzis *et al*.^18^. They found that energetics of imbibition compounds are involved in temperature rise and result in electrostatic attractions as the liquid molecules are adhered on the fiber surfaces upon capillary contact.

A higher temperature rise was observed for cellulose-based samples than the plastic-based paper. For a certain paper type, thicker and more layers of paper resulted in higher temperature rise. Addition of salt to the water caused a reduction of the temperature rise. All of these observations are in line with postulation that the energy released as a result of wetting of surface of paper fibers is the source of generated heat that causes local and temporary temperature rise.

## Electronic supplementary material

Supplementary Information
