# Supplementary figures and images for: Occurrence of temperature spikes at a wetting front during spontaneous imbibition
Source: Sci Rep. 2017 Aug 4;7:7268. doi: 10.1038/s41598-017-07528-7 (PMC5544723; doi:10.1038/s41598-017-07528-7)

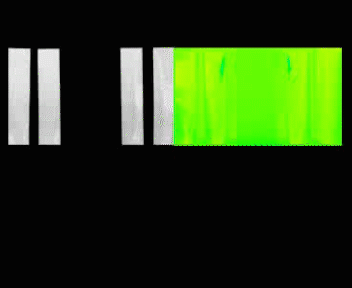

Supplement: Supplementary file 1 — Supplementary Information [file 41598_2017_7528_MOESM1_ESM.gif]
